# Efficacy and long-term follow-up of IL-1R inhibitor anakinra in adults with Still's disease: a case-series study

**DOI:** 10.1186/ar3366

**Published:** 2011-06-17

**Authors:** Katerina Laskari, Athanasios G Tzioufas, Haralampos M Moutsopoulos

**Affiliations:** 1 Department of Pathophysiology, School of Medicine, National and Kapodistrian University of Athens, Mikras Asias Street 75, Goudi 11527, Athens, Greece

## Abstract

**Introduction:**

To assess the efficacy and safety of the interleukin-1 receptor (IL-1R) inhibitor anakinra in adult patients with refractory Still's disease.

**Methods:**

Twenty-five patients (13 males and 12 females, median age 32 years, median disease duration seven months) with Still's disease were treated with subcutaneous injections of anakinra (100 mg/day). Treatment was given as adjunct therapy in 16 patients and as standalone in 9 patients for a median time of 15 months (range 1.5-71). The clinical and laboratory parameters during follow-up were recorded.

**Results:**

In 84% of patients the clinical activity resolved completely within a few days (median time 0.2 months), and response was maintained until the last visit in all but one patient. A complete response of all disease-related symptoms (clinical and laboratory) occurred subsequently within a median time of three months in 80% of patients. A partial clinical and laboratory improvement was shown in 12% and 16% of patients, respectively. The Visualized Analogue Scale and Health Assessment Questionnaire scores significantly decreased during treatment. The proportion of patients achieving the American College of Rheumatology 20 (ACR20) score (20% improvement) was 82% at one month and improved to 100% at one year. The mean oral corticosteroid dose was significantly reduced at each visit. Anakinra was discontinued due to unresponsiveness in one patient and due to relapsing disease in another. Treatment was also withdrawn in three patients with severe skin reactions (urticaria). Seven patients experienced an infection during follow-up.

**Conclusions:**

The rapid and sustained response in the majority of our patients encourages the use of anakinra in adults with Still's disease.

## Introduction

Still's disease is a rare systemic inflammatory disorder of unknown etiology characterized by quotidian spiking fevers, evanescent rash, arthritis, and elevated acute-phase reactants in the serum linked to alterations in the production of proinflammatory cytokines. Treatment has been empirical, with data on treatment efficacy extrapolated from case reports and small scale retrospective studies. Non-steroidal anti-inflammatory drugs and corticosteroids have been a mainstay for decades in treating adults with Still's disease; however, resistance to treatment as well as drug-related complications have been observed [1,2]. Immunosuppressants, mainly methotrexate (MTX), used as steroid-sparing drugs, have shown modest efficacy across studies [3,4]. In recent years, advances in the understanding of the role of cytokines in the disease pathogenesis has led to the application of anticytokine agents, often in combination with traditional immunosuppressive drugs, which opened a new, promising horizon in the treatment of adult Still's disease. Hence, TNF-a inhibitors have been successfully employed in some cases unresponsive to conventional treatment [5-10]. More recently, IL-1 activity has been linked to the pathogenesis of Still's disease as well as other systemic inflammatory diseases characterized in part by recurrent fevers, leucocytosis, anemia, and elevated acute-phase proteins, because rapid and sustained resolution of systemic and local inflammation is observed upon specific blockade of IL-1 receptors (IL-1R) [11]. The application of biological therapy with the IL-1R inhibitor anakinra, a recombinant form of the human IL-1R antagonist that inhibits IL-1 binding on its receptor approved for use in rheumatoid arthritis, appears to be promising treatment in patietns with refractory Still's disease [2,12-19].

## Materials and methods

### Patient selection and follow up

Twenty-five patients with active Still's disease (4 had juvenile onset and 21 adolescent- or adult-onset disease) resistant to glucocorticosteroids (n = 17), disease-modifying anti-rheumatic drugs (DMARDs; n = 4), or TNFa inhibitors (n = 4) who received treatment with anakinra were studied. All patients were diagnosed and followed in one medical center. All patients fulfilled the Yamaguchi disease criteria [20]. Patients younger than 18 years old were excluded from the study.

Information on patients was initially retrieved retrospectively from their medical records. Data were collected just before the initiation of anakinra treatment, at 1, 3, 6, and 12 months and at the latest follow up. Data included age, sex, disease duration, number of fulfilled Yamaguchi disease criteria, immunological profile, systemic symptoms (fever, rash, lymphadenopathy, hepatosplenomegaly, serositis, sore throat), tender joint count, swollen joint count, haematological profile, erythrocyte sedimentation rate (ESR), C-reactive protein (CRP), liver enzymes, and serum ferritin levels during follow up. The American College of Rheumatology (ACR) 20, 50, and 70 score (20%, 50%, and 70% improvement in joint disease, respectively) was also recorded [21]. Moreover, drug-related adverse events were recorded. After the relevant information was collected, each patient was contacted to have a final evaluation, performed for all cases by the same physician. Using the information retrieved from both the medical charts and the oral evaluation, the exact time-points of partial and complete response as well as relapse were determined. The Health Assessment Questionnaire (HAQ), Visual Analogue Scale (VAS) for the assessment of pain and global patient well-being, as well as VAS physician scores (physician assessment of disease activity) during follow up were calculated for each patient. Oral informed consent was obtained in all cases before starting treatment and the treatment regimen was approved by the hospital ethical committee.

### Assessments and definitions

The effect of anakinra on all clinical and laboratory disease-related manifestations during follow up was evaluated and drug adverse events were recorded. Moreover, the proportion of patients with complete clinical response (CCR) or complete laboratory response (CLR) and partial clinical response (PCR) or partial laboratory response (PLR) as well as the time to these events were determined. Complete response was defined as the complete resolution of all disease-related symptoms, except for joint erosion [18]. PCR or PLR was defined as improvement (at least 10% when measurement was feasible) in one or more related clinical or laboratory, respectively, manifestations, but without complete resolution of disease activity. Relapse was defined as a worsening in disease activity requiring switching to another treatment agent or the addition of another therapy to the underlying medication.

### Immunosuppressive treatment

Sixteen of the 25 patients received daily injections of anakinra (100 mg/day) subcutaneously as adjunct therapy together with a DMARD and nine patients received anakinra as monotherapy. Concomitant medication included MTX in 13 patients at a median (range) dose of 12.5 (7.5 to 20) mg/week, leflunomide at a low dose (10 mg/day) in one patient, and cyclosporine A at the dose of 3 mg/kg/day in another one. MTX was given during the whole follow up in 10 of the 13 patients. In three patients, MTX was discontinued eight, five, and five months after remission was achieved. In three other patients, MTX was added to treatment with anakinra later during follow up. Two of them started MTX at a disease flare, while the third one was in remission at MTX initiation. The two patients treated with either cyclosporine A or leflunomide as concomitant medication had a short follow up of 1.5 and 3 months. The oral methylprednisolone dose was slowly tapered during follow up based on the physician's assessment of disease activity.

### Statistical analysis

Scaled and/or ordinal patient characteristics were compared during follow up using the Wilcoxon test for paired observations and nominal parameters using the McNemar test. Time to event analyses were performed according to the Kaplan-Meier method. Disease outcome was compared between patients receiving and those not receiving concomitant medication using both the chi squared test and survival analysis in means of the log-rank test. Results were considered significant when *P *value of 0.05 or less. Analyses were conducted in SPSS version 13 (Chicago, Illinois, USA). All *P *values are two-tailed.

## Results

### Patient characteristics

Twenty-five patients (13 males and 12 females) received anakinra as adjunct therapy (n = 16) or monotherapy (n = 9) for a median (range) time of 15 (1.5 to 71) months. The median patient age was 32 (18 to 71) years. The median disease duration until anakinra treatment initiation was 7 (1 to 228) months and the median number of Yamaguchi disease criteria fulfilled at baseline was 6 (5 to 8). Three patients were positive for antinuclear antibodies and one patient developed rheumatoid factor positivity during follow up. Oral methylprednisolone was given to all but three patients at a median dose of 4 (range 0 to 26) mg/day. The median corticosteroid dose was significantly reduced at each visit (Table 1) and 12 patients were able to wean from steroids until the end of follow up.

**Table 1:** Patient clinical and laboratory characteristics during follow up

	At baseline (n = 25)	At 1 month * (n = 25)	At 3 months ** (n = 24)	At 6 months (n = 20)	At 12 months (n = 18)	At latest follow up (n = 15)
Fever	24 (96)	1 (4)	1 (4)	2 (10)	0	0
Rash	16 (64)	0	0	1 (5)	0	0
Lymphadenopathy	14 (56)	0	0	1 (5)	0	0
Arthralgias	22 (88)	9 (36)	5 (21)	2 (10)	0	0
TJC	12 (0-38)	0 (0-24)	0 (0-24)	0 (0-34)	NA ***	NA ***
Arthritis	15 (60)	2 (8)	2 (8)	2 (10)	0	0
SJC	1 (0-15)	0 (0-5)	0 (0-5)	0 (0-34)	NA ***	NA ***
Hepatosplenomegaly	11 (44)	0	0	1 (5)	0	0
Sore throat	14 (56)	1 (4)	1 (4)	1 (5)	0	0
Serositis	5 (20)	0	0	0	0	0
Anemia ‡ (Hb < 12 g/dl for females and < 13.5 g/dl for males)	16 (64)	10 (40)	5 (21)	2 (10)	0	0
Hb (g/dl)	11.8 (8.5-14.4)	12.7 (8.7-16)	13.1 (9.8-15.3)	14 (10-16)	13.7 (12-15.8)	14 (12.3-15.4)
Leucocytosis (> 10,000/mm^3^)	21 (84)	9 (36)	5 (21)	3 (15)	0	0
WBC (× 10^3^/mm^3^)	16.8 (4.8-33.6)	8.2 (3-20.6)	6.9 (2.8-15)	6.5 (3.5-18.4)	6.3 (3.2-9.8)	6.5 (4-9)
Thrombocytosis (> 450,000/mm^3^)	8 (32)	3 (12)	2 (8)	1 (5)	0	0
PLTs (× 10^3^/mm^3^)	378 (134-554)	228 (156-555)	251 (160-556)	215 (165-511)	236 (145-334)	210 (121-274)
Elevated CRP (> 10 mg/dl)	25 (100)	8 (32)	6 (25)	4 (20)	0	0
CRP (mg/dl)	111 (19-318)	5.4 (0.1-200)	6 (0.2-107)	3.6 (0.8-119)	2 (0.1-10)	3.5 (0.4-9)
Elevated ESR (> 30 mm/h)	24 (96)	6 (24)	2 (8)	1 (5)	0	0
ESR (mm/h)	75 (26-120)	20 (2-130)	11.5 (2-72)	10 (2-105)	6 (2-23)	4 (1-15)
High ferritin levels (> 300 ng/ml)	24 (96)	7 (28)	4 (17)	3 (15)	0	0
Ferritin (ng/ml)	3000 (192-88126)	217 (47-6413)	111 (24-7700)	165 (9-3163)	59 (10-209)	58 (41-124)
Elevated liver enzymes	15 (60)	6 (24)	1 (4)	2 (10)	2 (11)	0
HAQ	2.19 (0.25-3)	0.31 (0-2.25)	0 (0-1.44)	0 (0-3)	0 (0-1.25)	0 (0-1.25)
VAS pain	2.4 (0.6-3)	0 (0-2.4)	0 (0-2.7)	0 (0-2.7)	0 (0-1.5)	0 (0-1.5)
VAS global	2.25 (0.9-3)	0.3 (0-2.4)	0 (0-2.7)	0 (0-2.7)	0 (0-1.5)	0 (0-1.5)
VAS physician	1.8 (0.9-2.7)	0.3 (0-1.8)	0 (0-2.4)	0 (0-2.4)	0 (0-0.3)	0 (0-0.3)
ACR20	NA ***	18/22 (82)	17/21 (81)	15/17 (88)	15/15 (100)	13/13 (100)
ACR50	NA ***	15/22 (68)	16/21 (76)	15/17 (88)	14/15 (93)	13/13 (100)
ACR70	NA ***	14/22 (64)	15/21 (71)	13/17 (76)	13/15 (87)	12/13 (92)
Methylprednisolone oral dose † (mg/d)	18 (0-48)	14 (0-40)	8 (0-32)	7 (0-24)	0.5 (0-14)	0 (0-8)

* Significant comparisons of clinical and laboratory parameters at one month vs. baseline: fever *P *< 0.001, rash *P *< 0.001, lymphadenopathy *P *< 0.001, arthralgias *P *< 0.001, TJC *P *< 0.001, arthritis *P *< 0.001, SJC *P *= 0.01, hepatosplenomegaly *P *= 0.002, sore throat *P *< 0.001, serositis *P *= 0.063, Hb (g/dl) 0.007, leucocytosis *P *= 0.001, WBC (× 10^3^/mm^3^) *P *< 0.001, thrombocytopenia *P *= 0.063, PLTs (× 10^3^/mm^3^) *P *= 0.03, elevated CRP *P *< 0.001, CRP (mg/dl) *P *= 0.001, elevated ESR *P *< 0.001, ESR (mm/h) *P *= 0.013, high ferritin levels *P *= 0.004, ferritin (ng/ml) *P *= 0.002, elevated liver enzymes *P *= 0.021, HAQ score *P *< 0.001, VAS pain score *P *< 0.001, VAS global score *P *< 0.001, VAS physician score *P *< 0.001.

‡ Anemia at one month vs. baseline *P *= 0.375, at three months vs. baseline *P *= 0.008.

** Significant comparisons at three months vs. one month: CRP (mg/dl) *P *= 0.028, ESR (mm/h) *P *= 0.04, Hb (g/dl) *P *= 0.042.

† Significant reduction of the oral methylprednisolone dose during follow-up: at one month vs. baseline *P *= 0.001, at three months vs. one month *P *= 0.001, at six months vs. three months *P *= 0.015, at 12 months vs. six months *P *= 0.005, at end vs. 12 months *P *= 0.043.

*** NA, not applicable

ACR, American College of Rheumatology; CRP, C-reactive protein; ESR, erythrocyte sedimentation rate; HAQ, health assessment questionnaire; Hb, hemoglobin; PLTs, platelets; SJC, swollen joint count; TJC, tender joint count; VAS, visual analogue scale; WBC, white blood cells.

### Clinical and laboratory parameters during follow up

**Figure 1:**
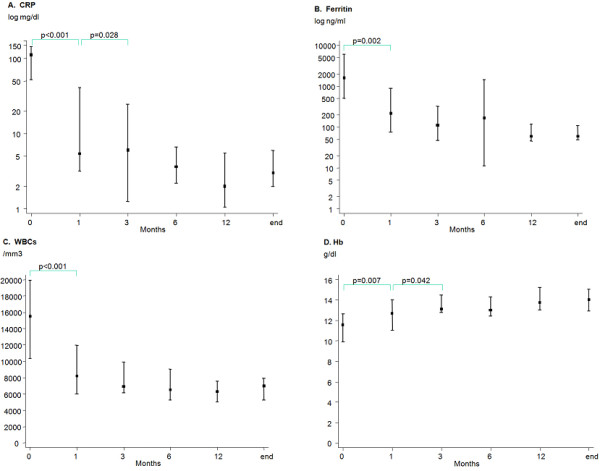
**Graphical presentation of the median values and 25^th^/75^th ^percentiles of (a) CRP levels, (b) ferritin levels, (c) WBC count, and (d) Hb levels during follow up**. In a and b the logarithmic values of C-reactive protein (CRP) and ferritin levels are provided. Hb, hemoglobin; WBCs, white blood cells.

The patient clinical and laboratory characteristics from baseline until the latest visit are shown in Table 1. Of the total 25 patients, 20 (80%) had a follow-up time of at least 6 months and 15 patients (60%) had a follow-up time greater than 1 year as shown in Table 1. A significant improvement in all investigated parameters was observed within the first month of treatment (Table 1 and Figure 1) and at each evaluation during follow up compared with baseline (*P *values not shown). When subsequent timepoints were compared with each other, no significant changes were observed (data not shown) except for hemoglobin, CRP, and ESR levels which continued to significantly improve after the first and up to the third month of treatment (Table 1). Although the hemoglobin levels significantly increased within the first month of treatment, the number of patients with anemia significantly decreased at three months (Table 1). A significant decrease in HAQ, and VAS pain, global and physician scores was demonstrated within the first month of treatment (Table 1). The percent of patients achieving the ACR20 score was 82% at one month and improved to 100% at one year. The ACR50 and ACR70 scores at one year were 93% and 87%, respectively. None of the patients presented the hemophagocytic syndrome complicating Still's disease. Renal amyloidosis was suspected in one patient with proteinuria (1.5 g in 24-hour urine collection), absence of active urine sediment, normal renal function, and absence of other underlying medical conditions that might cause renal damage. The amount of proteinuria did not improve during follow up, although other clinical and laboratory parameters normalized until the latest visit. None of the patients died.

### Complete and partial clinical response

In 21 (84%) of the 25 patients, clinical activity resolved completely and response was maintained until the last visit in all but one. Three (12%) of the 25 patients had partial response and presented with arthralgias (n = 2) or arthritis and intermittent fever (n = 1) at the end of follow up. One patient (4%) with prominent articular disease did not show any improvement within four months, was assigned to another treatment, and was withdrawn from the study (Patient 6, Table 2). In all but one patient with CCR, laboratory parameters were normal (Patient 5, Table 2). In contrast, all patients with PCR had persisting biological activity (Table 2). In survival analysis, a complete resolution of clinical activity was observed within a few days of the first injection in most patients (median time 0.2 months, range 0.01 to 8 months), while PCR was achieved within a few days (0.01 month) in one patient and at one month in the other two.

**Table 2:** Characteristics of patients with inadequate response to treatment or relapse

Pt	Age	HAQ at t0	Months of treatment/withdrawal	DMARD/mean GC oral dose (mg/d)	Previous treatment	PCR/CCR (months to event)	Persisting clinical symptoms	PLR/CLR (months to event)	Persisting biological activity	Relapse (months to event)
1	24	3	3/drug toxicity	CyA/16	GC	PCR (1)	Arthralgias	PLR (1)	↑ WBC, ↑ Ferritin, ↑ Liver enzymes	No
2	16	1.25	6/disease flare	MTX/8	MTX+GC +ETN	PCR (1)	Fever, Arthritis	PLR (1)	Anemia, ↑ WBC, ↑ PLTs, ↑ CRP, ↑ ESR, ↑ Ferritin	Yes (4)
3	56	2.5	3/drug toxicity	None/0	GC	PCR (0.01)	Arthralgias	PLR (1)	Anemia, ↑ Ferritin	No
4	64	1.5	1.5/drug toxicity	LEF/3	MTX+GC	CCR (1)	-	PLR (1)	Anemia, ↑ ESR	No
5	48	2.5	14/follow up end	MTX for the last 8 months/18.25	GC	PCR (0.5)/CCR (8)	Arthralgias/Arthritis	PLR (1)/CLR (9)	↑ WBC, ↑ CRP	Yes (5)
6	32	1.8	4/no response	MTX/4	MTX+GC +ETN, then ADA	No response	Arthralgias/Arthritis	No response	Anemia, ↑ WBC, ↑ PLTs, ↑ CRP, ↑ ESR, ↑ Ferritin	-
7	49	3	15/follow up end	MTX for the last 2 months/18.6	GC	CCR (0.2 and 13.5)	-	CLR (1 and 14)	-	Yes (13 e.g. 4 months after reducing anakinra dosing)

ADA, adalimumab; CCR, complete clinical response; CLR, complete laboratory response; CRP, C-reactive protein; CyA, cyclosporine A; DMARD, disease-modifying anti-rheumatic drug; ESR, erythrocyte sedimentation rate; ETN, etanercept; F, female; GC, glucocorticosteroids; HAQ, health assessment questionnaire; LEF, leflunomide; M, male; MTX, methotrexate; PCR, partial clinical response; PLR, partial laboratory response; PLTs, platelets; WBC, white blood cells.

### Complete and partial laboratory response

At the end of follow up, in 20 (80%) of the 25 patients laboratory parameters returned to normal, 4 (16%) showed a partial response, whereas 1 patient (4%) remained with active disease (Table 2). Disease was clinically silent in all patients with CLR. At the end of follow up, high ferritin levels and anemia were most commonly found among the five patients with persisting biological activity. More specifically, persisting abnormalities included high ferritin levels (n = 4), anemia (n = 4), leucocytosis (n = 3), thrombocytosis (n = 2), high CRP levels (n = 2), elevated ESR (n = 3), and elevated liver enzymes (n = 2; Table 2). All but one patient with serological activity were also clinically active (Patient 4, Table 2). A complete response of all biological disease-related symptoms occurred within a median time of 3 months (range 1 to 10 months), whereas a partial improvement in laboratory parameters was observed in the first month of treatment in all four patients with PLR at the end of follow up.

### Relapse

A disease exacerbation occurred in three patients (12%). One patient in PCR and PLR groups deteriorated at four months of treatment. The interval between spiking fever episodes decreased to daily events and articular involvement worsened (Patient 2, Table 2). Two months later immunosuppressive treatment was switched to another drug agent and the patient was withdrawn from the study. In another patient in PCR and PLR, the fever and rash reappeared, while articular involvement deteriorated five months after treatment initiation. In parallel, white blood cell (WBC) count, ferritin levels, and liver enzymes increased. MTX was started in addition to anakinra at a mean dose of 18.75 mg/week and the patient went into complete clinical and laboratory remission three and four months later, respectively (Patient 5, Table 2). A third patient, in complete remission of all disease-related manifestations for nine months, reduced anakinra to alternate day dosing and presented fever, rash, leucocytosis, and elevation of acute-phase reactants four months later. He was started on daily injections again combined with MTX 15 mg/week and went into complete disease remission within one month (Patient 7, Table 2).

### Adverse events

Three patients developed a severe urticarial reaction after the first months of treatment (one patient at 1.5 month and two patients at 3 months) and discontinued therapy. Seven patients (28%) developed infections (one trachiobronchitis, one H1N1 virus infection of the upper respiratory tract, one gastroenteritis with fever, one soft tissue abscess, and three lower urinary tract infections), which led to a transient discontinuation of the immunosuppressive treatment. The transient withdrawal of anakinra led to the recurrence of fever and arthralgias in one patient; however, symptoms automatically resolved after drug reinstitution. One patient receiving MTX as concomitant medication had a slight increase in liver enzymes later during follow up (less than a two-fold increase compared with normal values) with no sign of disease flare considered as drug toxicity. Five patients had a local hypersensitivity reaction at the site of injection.

### Anakinra as adjunct therapy vs. monotherapy

When disease outcome was compared between patients receiving and those not receiving concomitant medication with a DMARD, no significant differences were demonstrated in the achievement of CCR, PCR, CLR, PLR, and relapse both in means of the chi squared test (*P *= 1.00) and survival analysis (*P*≥0.10 in all comparisons; data not shown). Moreover, the number of tender and swollen joints at baseline and during follow up were comparable (*P *> 0.30 in all comparisons). The number of drug-related adverse events did not significantly differ between patients treated with a DMARD and those who received anakinra as monotherapy (*P *= 1.00).

### Anakinra dosing reduction and long-term follow up

Seven of the 20 patients in complete disease remission increased the interval between anakinra injections by receiving one dose every other day during the last months of follow up (median duration 6 months, range 4 to 30 months). The patients were in remission for a median time of 12 months (7 to 25 months) before anakinra dosing tapering. Only one of these patients relapsed four months after anakinra dosing reduction (see paragraph about relapse). In eight patients in complete remission (including three of the above patients) treatment was discontinued. Two of them relapsed one month after treatment withdrawal, whereas the remaining six are still in remission after a median time period of 6.5 months (1 to 60 months) of treatment discontinuation.

## Discussion

In the present study we assessed the efficacy and safety of the IL-1R inhibitor anakinra in adult patients with refractory Still's disease. To our knowledge, this is the largest series reported in literature, because previous reports consist of smaller patient series as well as case reports [2,13-19,22].

In agreement with previous data, we observed a rapid and sustained response to anakinra treatment in the majority of patients [2,13-19]. A dramatic improvement in all disease-related manifestations was evident from the first month of treatment. Complete resolution of clinical symptoms occurred within a few days after the initiation of treatment and was followed by normalization of acute-phase reactants, hematological parameters, and biochemical markers of systemic disease within the first three months. According to the already reported literature, the time to complete resolution of clinical activity varied from a few hours after the first injection up to two months of treatment, with most cases remitting within some days, while laboratory markers returned to normal subsequently within one week to two months [13-19]. In the two largest already published series of 15 and 8 patients, respectively, disease activity completely resolved in 60% and 90% of patients, respectively, whereas no efficacy was reported in 13% of patients in the first study [18,19]. In accordance with our data, persisting clinical activity was expressed with arthralgias or fever. Elevated CRP, ESR, and ferritin levels were the abnormal laboratory markers in patients with residual serological activity. In line with our observations, Maier et al. reported anemia and high ferritin levels as disease markers that normalized at last [17]. In our study, clinical activity resolved completely in 84% and laboratory parameters returned to normal in 80% of patients, while 4% did not show any response to treatment. In only two patients, anakinra was discontinued because of inadequate response. It is of note that three of four patients in partial response had a short follow-up time of 1.5 to 3 months because of an early drug withdrawal. Thus, a potential delayed response in those patients if they were treated for a longer time period cannot be excluded.

The great efficacy of the IL-1R inhibitor points toward the important role of IL-1 in the pathophysiological processes underlying Still's disease. Indeed, increased IL-1 levels have been detected in active untreated disease [13,15]. Kötter et al demonstrated high IL-1 and IL-18 in active disease, whereas moderately high TNF-a and IL-6 levels, that significantly fell following treatment with the IL-1R inhibitor [15]. Similarly, Fitzgerald et al. demonstrated high IL-1a, IL-1b, IL-1RA, IL-6, and IL-18 serum levels prior to the first injection of anakinra, while a dramatic reduction in the levels of IL-6, IL-18, and IL-1RA accompanied the disease activity resolution upon treatment initiation [13]. An increase in the serum proinflammatory cytokine levels was observed when therapy was stopped. However, the group of partial responders and non-responders might be suggestive of disease subtype heterogeneity and likely reflects different roles of cytokines and pathological processes in these patients [23,24]. In fact, one patient in the present study with prominent articular involvement without clinical signs of systemic disease, did not improve during anakinra treatment.

Relapses under anakinra are rarely reported [13,19]. However, studies with a large number of patients and a long period of follow up are still pending. In this report, the effects of anakinra treatment were sustained until the latest visit in all but one complete responder. This patient relapsed after reducing the frequency of drug injections. Two other patients who experienced a disease flare were in partial response.

There is no clear evidence whether the use of anakinra in combination with a DMARD, such as MTX, improves its efficacy. In our series, either the overall efficacy or the articular disease were both comparable between patients receiving anakinra as adjunct and those as monotherapy. Similarly, in the report by Lequerré et al, DMARD use was not particularly different in the complete response vs. the non-responder cohort [18]. However, long-term data are missing. Our study provides evidence that anakinra monotherapy may be an effective therapeutic option in some patients, whereas combination with a DMARD may be necessary when residual or recurrent disease activity is present. In fact, recurrent disease in the form of arthralgias/arthritis, fever, rash, and acute inflammatory response was effectively controlled by the addition of MTX to anakinra treatment, as also previously reported [13,19].

Regarding concomitant oral corticosteroid usage, anakinra was shown to be an effective steroid-sparing agent. In agreement with the already reported literature, the use of anakinra allowed fast steroid tapering in responders and total discontinuation of steroid intake in approximately half of them [13,14,17-19]. Moreover, four patients did not require any corticosteroid therapy during the whole follow up.

The optimal treatment duration with anakinra is unknown. An interesting issue is whether the number of anakinra injections per week can be decreased in responders. Gradual reduction of anakinra dosing after a median of 12 months on remission seemed to be safe in our series, because only one of seven patients under alternate day treatment relapsed. Safe increasing of the time interval between anakinra applications has been also reported by Maier et al., whereas Kötter et al. described recurrence of disease activity after reducing anakinra to alternate day dosing [15,17]. In responders, total drug discontinuation led to the recurrence of disease activity in two of eight of our patients, while the rest of patients remained in complete remission for a median time of 6.5 months. Thus, remission may be maintained in some patients after drug discontinuation. However, given the small number of patients and the relatively short follow up after drug withdrawal, any conclusions can only be interpreted with caution.

In regard to toxicity, anakinra was safe and well tolerated in all but three patients who discontinued therapy because of severe urticarial-like skin reactions and one patient with severe trachiobronchitis. Lequerré et al. reported anakinra withdrawal in two patients who developed skin rash at first and third month of treatment, whereas severe systemic inflammatory response syndrome has been also described as an IL-RA therapy complication [18,22]. Infections were not rare in our patients (28%). The observed rates were similar with those described previously [18]. Finally, local pain or reactions to injections have been frequently reported in literature [15,16,18,19]. The rebound of disease activity following transient cessation of IL-1RA therapy due to opportunistic infections, was noticed in one of our patients as well as in other published cases; however, disease activity resolved after drug reinstitution in all of them [13,16,19].

Compared with TNFa inhibitors or MTX monotherapy, anakinra seems to have a more rapid and efficient effect in adult patients with Still's disease. Response rates in the largest series using anti-TNFs and/or MTX were lower than in the present study ranging between 25% and 69%, whereas worsening of disease and/or exacerbations were also reported [3-6]. Compared with anakinra, the response was delayed with most cases remitting after two weeks of treatment and up to 16 weeks. However, most promising results have been reported in smaller series [9,10]. At this point, it is worth presenting unpublished data on a historical control of adult patients with Still's disease followed up in our department and treated with TNF inhibitors (infliximab n = 4, etanercept n = 2, adalimumab n = 1), and (n = 5) or MTX (n = 6). In accordance with previously reported data, 54% of patients went into complete disease remission, whereas 43% of the responders relapsed. The median time to clinical response was four months (range 0.5 to 6 months), while laboratory parameters returned to normal after a median time of four months (range 0.5 to 10 months). Sequential treatment with different anti-TNFs in five of our patients did not change drug efficacy, whereas two of four patients who received anakinra due to unresponsiveness to anti-TNFs went into remission.

Finally, our results should be interpreted in the context of potential limitations. The present study was not prospectively designed. Therefore, detailed information on the drug use before the initiation of anakinra was not always available. Moreover, the relatively small number of patients limits the statistical power of the study. Thus, no definite conclusions can be drawn regarding the efficacy of anakinra as adjunct vs. monotherapy. Additionally, more than one third of our cohort had a duration of follow up shorter than one year. Nevertheless, the rapid clinical response as well as the long-lasting efficacy together with the steroid-sparing effect and the favorable safety profile in most of our patients encourages further use of anakinra in adults with Still's disease.

## Conclusions

Based on these observations, we suggest that blocking IL-1R as monotherapy or in combination with a DMARD may become the treatment of choice for patients with refractory Still's disease.

## Abbreviations

ACR: American College of Rheumatology; CCR: complete clinical response; CLR: complete laboratory response; CRP: C-reactive protein; DMARD: disease-modifying anti-rheumatic drug; ESR: erythrocyte sedimentation rate; HAQ: health assessment questionnaire; IL: interleukin; IL-1R: interleukin-1 receptor; MTX: methotrexate; PCR: partial clinical response; PLR: partial laboratory response; TNF-a: tumor necrosis factor-a; VAS: visualized analogue scale; WBCs: white blood cells.

## Competing interests

The authors declare that they have no competing interests.

## Authors' contributions

KL participated in the design of the study, collected the data, performed the statistical analysis and interpretation of data, and drafted the article. AGT participated in the design of the study and its coordination, helped in revising the article, and provided intellectual content of critical importance. HMM conceived of the study, and provided intellectual content of critical importance. All authors read and approved the final manuscript.

## Authors' information

KL, MD: Resident in Rheumatology at the Department of Pathophysiology, School of Medicine, University of Athens, Athens, Greece

AGT, MD: Professor at the Department of Pathophysiology, School of Medicine, University of Athens, Athens, Greece

HMM, MD, FRCP, FACP, Master ACR: Professor at the Department of Pathophysiology, School of Medicine, University of Athens, Athens, Greece
